# Intraductal Papillary Neoplasms of the Bile Duct

**DOI:** 10.1155/2014/459091

**Published:** 2014-05-18

**Authors:** Masayuki Ohtsuka, Hiroaki Shimizu, Atsushi Kato, Hideyuki Yoshitomi, Katsunori Furukawa, Toshio Tsuyuguchi, Yuji Sakai, Osamu Yokosuka, Masaru Miyazaki

**Affiliations:** ^1^Department of General Surgery, Graduate School of Medicine, Chiba University, 1-8-1 Inohana, Chuoh-ku, Chiba 260-8670, Japan; ^2^Department of Medicine and Clinical Oncology, Graduate School of Medicine, Chiba University, Chiba 260-8670, Japan

## Abstract

Intraductal papillary neoplasm of the bile duct (IPNB) is a rare variant of bile duct tumors characterized by papillary growth within the bile duct lumen and is regarded as a biliary counterpart of intraductal papillary mucinous neoplasm of the pancreas. IPNBs display a spectrum of premalignant lesion towards invasive cholangiocarcinoma. The most common radiologic findings for IPNB are bile duct dilatation and intraductal masses. The major treatment of IPNB is surgical resection. Ultrasonography, computed tomography, magnetic resonance image, and cholangiography are usually performed to assess tumor location and extension. Cholangioscopy can confirm the histology and assess the extent of the tumor including superficial spreading along the biliary epithelium. However, pathologic diagnosis by preoperative biopsy cannot always reflect the maximum degree of atypia, because IPNBs are often composed of varying degrees of cytoarchitectural atypia. IPNBs are microscopically classified into four epithelial subtypes, such as pancreatobiliary, intestinal, gastric, and oncocytic types. Most cases of IPNB are IPN with high-grade intraepithelial neoplasia or with an associated invasive carcinoma. The histologic types of invasive lesions are either tubular adenocarcinoma or mucinous carcinoma. Although several authors have investigated molecular genetic changes during the development and progression of IPNB, these are still poorly characterized and controversial.

## 1. Introduction

Intraductal papillary neoplasm of the bile duct (IPNB) is a rare variant of bile duct tumors, which is characterized by papillary or villous growth within the bile duct lumen.

Formerly, attention has been drawn to biliary tumors with macroscopically visible mucin secretion, which show predominantly papillary growth within the dilated bile duct lumen and secrete a large amount of mucin. These tumors were called by various names, such as mucin-producing cholangiocarcinoma [1–4], mucin-hypersecreting bile duct tumor [5], and intraductal papillary mucinous tumor of the bile duct [6, 7], and were identified as a biliary counterpart of intraductal papillary mucinous neoplasm (IPMN) of the pancreas. On the other hand, biliary intraductal tumors without macroscopically visible mucin secretion are also known, which have a macroscopically recognizable papillary or granular structure but no clinically visible mucin secretion. Since certain morphological features of these tumors, especially intraductal papillary growth pattern, are also similar to those of IPMN of the pancreas, Zen et al. [8] proposed that they, together with tumors with macroscopically visible mucin secretion, may belong to a single tumor entity named IPNB. Now, IPNB was adopted in the 2010 World Health Organization (WHO) classification [9] as a distinct clinical and pathologic entity. In this review, we describe the concept, clinical and pathologic features, and pathogenesis of IPNB.

## 2. Concept of IPNB

### 2.1. Definition of IPNB

IPNB is defined as a biliary epithelial tumor with exophytic nature exhibiting papillary mass within the bile duct lumen and with prominent intraductal growth pattern. IPNB can develop anywhere along the biliary tree, including both intrahepatic and extrahepatic bile ducts. Mucin hypersecretion and dilatation of the bile duct are sometimes encountered. Microscopically, IPNB is composed of papillary fronds with fine vascular cores. Neoplastic epithelial cells display a spectrum of cytoarchitectural atypia ranging from none to borderline to marked and also can be associated with invasive carcinoma. Due to these features, IPNB is regarded as a premalignant lesion towards invasive cholangiocarcinoma. In the WHO classification [9], IPNB is classified into IPN with low- or intermediate-grade intraepithelial neoplasia, IPN with high-grade intraepithelial neoplasia, and IPN with an associated invasive carcinoma. This classification is similar to that of IPMN of the pancreas, and an analogous multistep progression model is assumed in IPNB.

### 2.2. Diseases Included in IPNB

Before inclusion of IPNB in the WHO classification, many different terms have been used for the spectrum of this entity. These include biliary papilloma/papillomatosis, some of the intraductal growth type of cholangiocarcinoma and papillary carcinoma of the extrahepatic bile duct, and some of the biliary cystadenoma/cystadenocarcinoma. Among the intraductal growth type of intrahepatic cholangiocarcinoma and papillary carcinoma of the extrahepatic bile duct, cases with intraductal component composed of papillary fronds with fine vascular cores are exclusively included in IPNB. In the previous categories of biliary cystadenoma/cystadenocarcinoma, cystic tumors with bile duct communication and absence of ovarian-like stroma are considered as a cystic variant of IPNB [10].

## 3. Clinical Features 

### 3.1. Clinical Characteristics

The prevalence of IPNB shows wide geographic variation. The highest incidence is reported in Far Eastern countries, probably because hepatolithiasis and clonorchiasis that are believed to be major risk factors of IPNB are endemic. IPNB is relatively rare and comprises 9–38% of all bile duct carcinomas [11–15]. Most patients are between 50 and 70 years of age [11–18] and show a slight male predominance in most reported series [12–14, 16–18]. Intermittent abdominal pain and acute cholangitis or jaundice are the most common clinical manifestations [11–13, 16, 18, 19], but certain frequency (5–29%) of patients have no symptoms [12, 13, 16, 18, 19]. Around 30% of patients have a previous history or concomitant existence of biliary stones, as shown in the reports from Far Eastern countries [12, 16, 20], but not from Western countries [13].

Tumor location varies by a report. Some reports showed that the majority of IPNB was located at the intrahepatic bile duct [16, 17], whereas the other showed that the most common location of IPNB was the hepatic hilum [13]. Despite these variable locations, IPNB tends to be found in the left-sided biliary ductal system, when IPNB exists in the intrahepatic bile duct, due to unknown reasons [13, 20, 21].

### 3.2. Radiologic Findings

**Figure 1:**
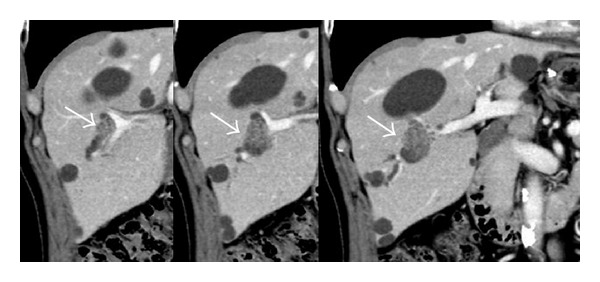
Representative images of intraductal papillary neoplasm of the bile duct on computed tomography. Localized bile duct dilatation and an intraductal mass are shown (arrows).

The most common radiologic findings for IPNB are bile duct dilatation and intraductal masses (Figure 1). The patterns of bile duct dilatation are diffuse duct ectasia, localized duct dilatation, and cystic dilatation, which can be recognized by ultrasonography (US), computed tomography (CT), and magnetic resonance image (MRI). These modalities can also detect intraductal masses, although its sensitivity is reported to be in the range of 41.2–97% [22–24]. MRI images reveal IPNB as iso- to hypointense masses on T1-weighted image and hyperintense masses on T2-weighted image [24]. The enhancement pattern on CT scan is isodense or hyperdense during the late arterial phase and not hyperdense during the portal-venous and delayed phase, as compared with normal hepatic parenchyma [23]. Mucin, even if it exists, cannot be detected on US, CT, and MRI.

**Figure 2:**
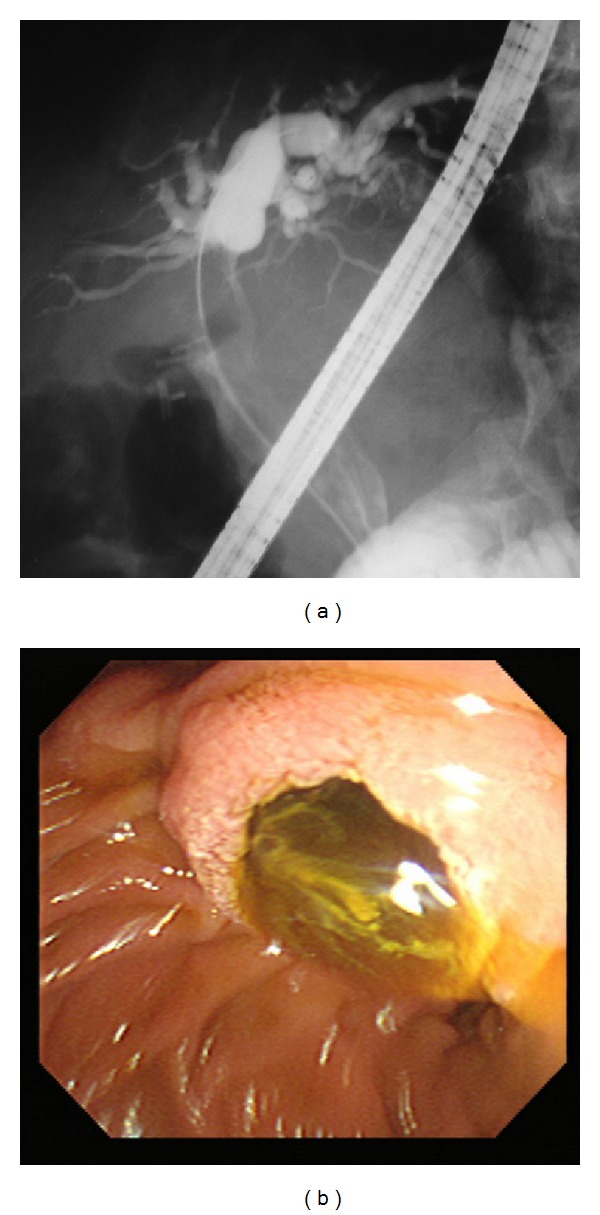
A representative case of intraductal papillary neoplasm of the bile duct with mucin hypersecretion. (a) Endoscopic retrograde cholangiogram. Diffuse dilatation of the common bile duct with amorphous filling defect is shown. (b) Duodenoscopy shows a dilated papillary orifice with mucin.

Direct cholangiography such as endoscopic retrograde cholangiography (ERC) is useful for the detection of mucobilia (Figure 2(a)) that is seen in nearly one-third of patients with IPNB, evidenced by diffuse dilatation of the bile duct with amorphous filling defect [6], and duodenoscopy shows a dilated papillary orifice with mucin (Figure 2(b)). However, the thick mucin that filled the dilated biliary tree often prevents the visualization of intraductal tumors [6, 25, 26]. In cases with IPNB without excessive mucin production, cholangiography can define the tumors as irregular filling defects.

**Figure 3:**
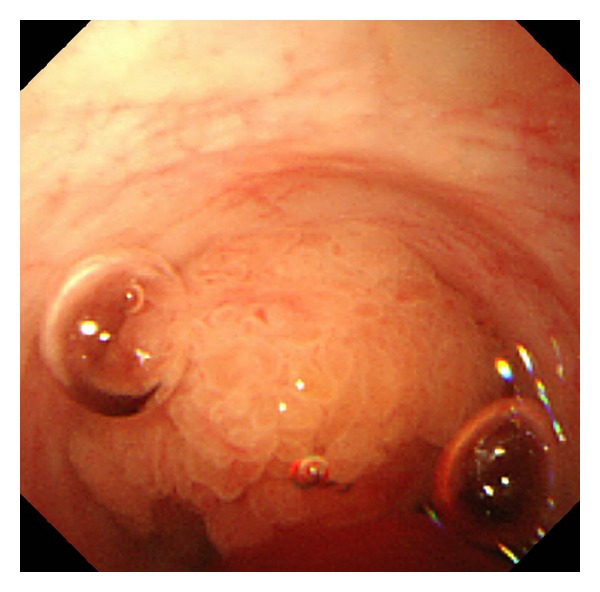
Peroral cholangioscopy reveals a papillary tumor within the lumen of the bile duct, but no obvious superficial spreading along the biliary epithelium is observed.

Cholangioscopy including percutaneous transhepatic cholangioscopy (PTCS) and peroral cholangioscopy (POCS) can approach the bile duct directly, and it can confirm the histology and assess the extent of the tumor including superficial spreading along the biliary epithelium (Figure 3), which provides information to choose appropriate treatment [22], although an accurate diagnosis of the maximum degree of cytoarchitectural atypia cannot be always made by biopsy because of the existence of mixed pathologic findings in the same lesion. POCS is advantageous in the fact that it can be performed without serious complications, such as catheter dislodgement, hemobilia, and tumor seeding of the sinus tract caused by PTCS [25, 27]. In cases with IPNB with abundant mucin, however, PTCS seems to be more useful than POCS, because discrimination of the location and extent of a tumor may be difficult by POCS in some cases [27].

Intraductal ultrasonography (IDUS) is a simple method for diagnosing the location of IPNB and assessing the depth of invasion, even in the presence of thick mucin. However, IDUS image is sometimes difficult to interpret, since coexisting biliary sludge may have an appearance like that of elevated tumors. Furthermore, it is difficult to distinguish between inflammatory wall thickness and the superficial spreading of a tumor [25].

### 3.3. Treatment

Unlike patients with IPMNs of the pancreas, all patients with IPNB should be considered to treat, because papillary tumors and associated mucin often cause recurrent cholangitis and obstructive jaundice, even if these tumors are not malignant. Patients without distant metastasis are considered for surgical resection. In order to choose appropriate surgical procedure, exact preoperative assessment of tumor location and extension is important. In particular, for evaluating of the extent of superficial spreading, cholangioscopic observation and biopsy might be essential. The depth of invasion and the presence of lymph node involvement are also assessed preoperatively by CT, cholangiography, and IDUS.

In principle, IPNBs should be resected in a manner similar to that employed for other types of intrahepatic cholangiocarcinomas and extrahepatic bile duct carcinomas. That is, major hepatectomy with or without extrahepatic bile duct resection or pancreaticoduodenectomy should be chosen as surgical procedure. Even though it is suspected that the tumor is premalignant, a similar strategy should be considered, because pathologic diagnosis by preoperative biopsy cannot always reflect the maximum degree of cytoarchitectural atypia. Intraoperative frozen section at the stumps of the bile duct is essential to confirm cancer-free surgical margin. Regional lymphadenectomy should also be performed.

On the other hand, in cases of IPNB with low- to high-grade intraepithelial neoplasia and limited superficial spreading and precise diagnosis which is completed preoperatively, limited resections preserving organ functions, for example, extensive hilar bile duct resection using a transhepatic approach [28, 29], can be considered as a choice among surgical procedures, although these should always be contingent on a careful intraoperative final assessment. In contrast, in cases of IPNB with extensive superficial spreading that may have positive margins or IPNB with multifocal involvement, tumor recurrence may occur with a high risk after surgical resection. In such cases, resection for the whole biliary tree by liver transplantation and pancreaticoduodenectomy can be theoretically regarded as the only curative treatment [30]. However, liver transplantation should not be performed in patients with advanced tumor invasion or with positive lymph nodes. Since accurate preoperative assessment of IPNB is usually difficult, indication of liver transplantation for patients with IPNB is very limited. 

## 4. Pathologic Features

### 4.1. Macroscopic Findings

**Figure 4:**
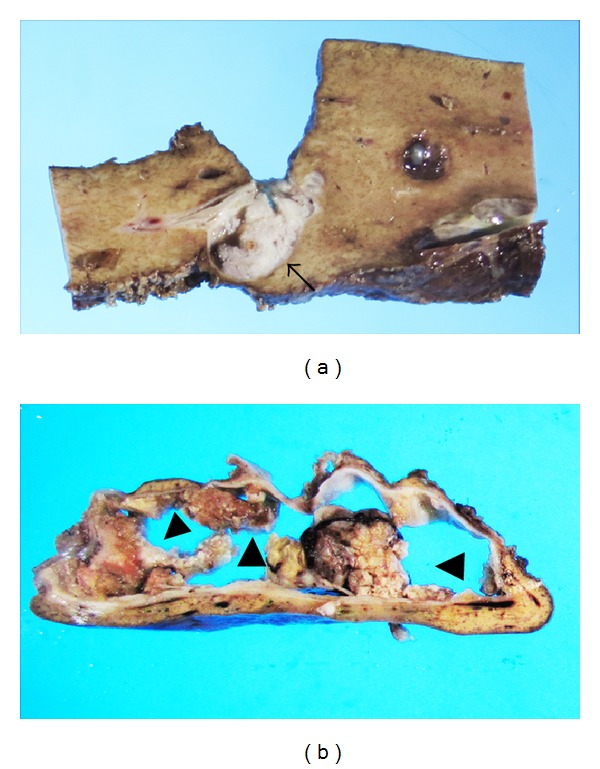
Macroscopic findings of intraductal papillary neoplasm of the bile duct. (a) A polypoid mass (arrow) is elevated into the lumen of the bile duct. (b) Polypoid mural nodules (arrowheads) are observed in the well-defined cystic lesion. This lesion was communicated with the bile duct.

The most common macroscopic findings of IPNB are singular, or occasionally multiple, polypoid masses elevating into the lumen of the dilated bile duct and/or clinically visible granular or small papillary mucosa (Figure 4(a)). Polypoid masses occasionally extend longitudinally and fill the lumen of the bile duct, showing cast-like appearance. Multilocular, rarely unilocular, well-defined cystic mass, which contains mucinous fluid, is another manifestation of IPNB (Figure 4(b)). The internal surfaces of cystic masses are generally smooth or finely granular, and papillary mural nodules are commonly observed. Anatomic communication with the bile duct is sometimes difficult to confirm.

### 4.2. Microscopic Findings

#### 4.2.1. Conventional Histology

**Figure 5:**
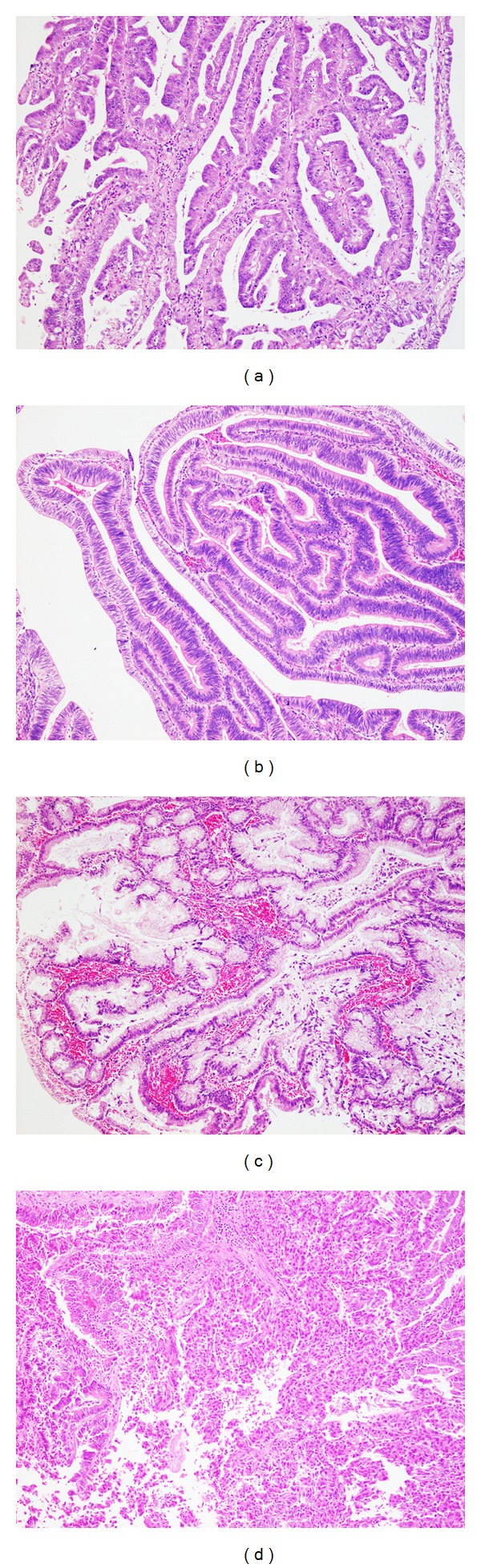
Microscopic findings of intraductal papillary neoplasm of the bile duct. Prominent papillary proliferation with delicate fibrovascular cores is a characteristic feature. Epithelial subtypes are classified as pancreatobiliary (a), intestinal (b), gastric (c), and oncocytic (d).

Prominent papillary proliferation with delicate fibrovascular cores is a characteristic finding (Figure 5). Coexistence of tubulopapillary architecture can be found in IPNB, especially without mucin hypersecretion [12]. Similar to IPMNs of the pancreas, IPNBs are classified into four epithelial subtypes (Figure 5), such as pancreatobiliary, intestinal, gastric, and oncocytic types, of the intraductal component [12–14, 16, 31]. The most frequent subtype is pancreatobiliary, followed by intestinal in all IPNBs, whereas IPNBs with mucin hypersecretion are more prevalent in the intestinal subtype than those without mucin hypersecretion [12]. The pancreatobiliary or the intestinal type is commonly associated with histologic grade of more than high-grade intraepithelial neoplasia, and, therefore, most cases of IPNB are IPN with high-grade intraepithelial neoplasia or IPN with an associated invasive carcinoma. The histologic types of invasive lesions are either tubular adenocarcinoma or mucinous (colloid) carcinoma [8]. Mucinous carcinoma usually arises in association with the intestinal type of IPNB.

IPNBs, however, often exhibited marked variation in histologic grade between different regions of individual tumors, making an accurate preoperative diagnosis difficult. This feature is significantly more common in IPNBs with mucin hypersecretion than those without [12].

IPNBs manifesting cystic mass have similar morphological features to biliary mucinous cystic neoplasms. These two entities are histologically distinct. Biliary mucinous cystic neoplasms have densely cellular connective tissue resembling ovarian stroma (ovarian-like stroma) in their wall, whereas this is never seen in IPNBs [10, 32].

#### 4.2.2. Immunohistochemical Phenotypes (Table 1)

Immunohistochemical mucin core proteins are reported to be associated with epithelial subtypes in IPMN of the pancreas. Similarly, MUC1 is often detected in the pancreatobiliary type of IPNBs, but very few are expressed in the intestinal or gastric type. MUC2 is primarily expressed in the intestinal type of IPNBs compared to the pancreatobiliary or the gastric type. MUC5AC expression is common in all epithelial subtypes, including the oncocytic type. In the oncocytic type of IPNBs, MUC1 expression is focally seen [16].

Some cytokeratin is also associated with epithelial subtypes. Cytokeratin 20 is expressed in the intestinal type of IPNBs with high frequency but not in the gastric type. High expression of cytokeratin 7 is observed in the gastric type of IPNBs [33].

## 5. Pathogenesis

### 5.1. Molecular Events during Development and Progression of IPNB (Tables 2 and 3) 

IPNBs derive from normal epithelium of the bile duct and progress through low-, intermediate-, and high-grade intraepithelial neoplasia to invasive carcinoma. During this process, cumulative aberrations in gene expression may be associated. However, these aberrations are still poorly characterized, and it is also not well known whether progression pathways of biliary intraepithelial neoplasia (BilIN), a precursor associated with the development of nonpapillary invasive cholangiocarcinoma, and IPNB are regulated differently. Several authors have investigated molecular genetic changes during the development and progression of the IPNB lineage and compared them with those of the BilIN lineage. According to the results in these studies mentioned below, IPNB and BilIN lineages were suggested to display a lot of similarities, but some differences, in the molecular genetic changes, although there were some inconsistent data among the reports.

Cyclins D1 and p21, which are the regulators of cell cycle progression, seem to play an important role in the development and progression in both BilIN and IPNB lineages, since expressions of these molecules have been reported to increase with histologic progression from low-grade to invasive carcinoma in both IPNBs and BilINs. Itatsu et al. [34] found that the positive rate of cyclin D1 expression in the IPNB lineage (65%) was significantly higher than that in the BilIN lineage (20%), suggesting that cyclin D1 is more important to the IPNB lineage, whereas Nakanishi et al. [35] have not shown such differences. Aberrant expression of p16, another regulator of cell cycle progression, was also seen from an early phase in the development of both BilIN and IPNB lineages, although the frequency of positive cases was relatively low, and the expression reached a plateau despite histologic progression [36, 37].

C-myc, which is a transcriptional factor for modulating regulators of cell cycle progression and a target molecule of Wnt signaling pathway, is suggested to be more important in the progression of the IPNB lineage than in that of the BilIN lineage. The expression of c-myc was demonstrated to be in more than half of IPNBs [34]. Similarly, nuclear accumulation of *β*-catenin protein, indicating genetic alteration of Wnt signaling pathway, was found only in approximately 25% of IPNBs [34, 38], concluding that this is significantly involved in the progression of IPNBs but not BilINs. However, a recent report has shown an inconsistent conclusion, in which *β*-catenin protein accumulation in the nucleus is less important for the progression of IPNBs due to its infrequency (9%) [36].

v-Ki-ras2 Kirsten rat sarcoma viral oncogene homolog (KRAS) mutations are indicated to be an early event in IPNBs, as shown by several reports [36, 38, 40–42]. The occurrence of these mutations was more common in IPNBs (17.6 to 46.2% of cases) than in BilINs. In contrast, with regard to guanine nucleotide-binding protein, *α*-stimulating activity polypeptide (GNAS) codon 201 mutations, which have been exclusively detected in approximately two-thirds of IPMNs of the pancreas but not pancreatic ductal adenocarcinoma [39], there are some conflicting data among the studies. Sasaki et al. [41] showed that GNAS mutation was detected in 15 of 30 IPNBs, whereas Schlitter et al. [36] and Matthaei et al. [40] found GNAS mutation only in one of 44 IPNBs and one of 23 IPNBs, respectively. Although reasons for this discrepancy are unknown, one possible reason may be difference of phenotypes of IPNBs studied. Tsai et al. [42] recently reported that 12 of 41 IPNBs showed GNAS mutation, which was correlated with a distinct subgroup of IPNB characterized by the intestinal subtype, villous configuration, and mucin hypersecretion. These features were extremely similar to those of IPMN of the pancreas. Similarly, all IPNBs with GNAS mutation only showed high-mucin production in the study by Sasaki et al. [41], whereas GNAS mutation was detected in the intestinal subtype in both studies by Schlitter et al. [36] and Matthaei et al. [40]. Furthermore, only one IPNB with mucin hypersecretion was included in the study by Schlitter et al. [36] and only two tumors with the intestinal subtype in the study by Matthaei et al. [40].

Involvement of SMAD4/DPC4, which acts as a tumor suppressor that functions in the regulation of the TGF-*β* signal transduction pathway, and p53, which acts also as a tumor suppressor, during the development and progression of IPNB is still controversial. Nakanishi et al. [35] showed that loss of SMAD4/DPC4 expression was seen in both IPNB (21.4%) and BilIN (27.3%) lineages with gradually increasing frequency with progression. Schlitter et al. [36] revealed similar results despite less frequency (IPNBs, 7%; BilINs, 14%). In contrast, Abraham et al. [38] reported that immunohistochemical labeling for SMAD4/DPC4 showed intact protein expression in all the IPNBs examined. One report [35] showed that aberrant immunohistochemical expression of p53 was early on in low-grade IPNB and reached a plateau, whereas that remained low in the early phase of BilIN lineage and its expression was significantly upregulated in the cases with invasive carcinoma. However, there were reports in which aberrant expression of p53 was never seen in all IPNBs examined [38], or p53 was not aberrantly expressed in IPNBs without invasion but extensively expressed in IPNBs with invasion [37]. Another report revealed that frequency of p53 aberrant expression progressively increased from low-grade intraepithelial neoplasia to invasive carcinoma [36].

There were few studies on DNA mismatch repair functionality in IPNBs. Abraham et al. [43] showed that impaired DNA mismatch repair evidenced by microsatellite instability was seen in 8 of 17 IPNBs (high-level in 2, low-level in 1). This frequency was higher than that previously reported for extrahepatic [44] and intrahepatic cholangiocarcinoma [45], indicating that impaired DNA mismatch repair might play a role in the pathogenesis of a subset of IPNBs. However, the mechanism that causes impaired DNA mismatch repair was not clarified, and no methylation of the human Mut L homologue gene promoter was detected in IPNBs.

Mucin core proteins such as MUC1 and MUC2 are involved in the progression of both IPNB and BilIN lineages. Zen et al. [46] reported that MUC1 expression was more common in BilINs, especially in invasive lesions, than in IPNB with an associated invasive carcinoma. They supposed two progression pathways of IPNB to tubular adenocarcinoma and mucinous carcinoma, featuring the phenotypes of MUC1+/MUC2+ and MUC1−/MUC2+, respectively, which are analogous to that of IPMN of the pancreas. However, Onoe et al. [14] revealed that most IPNB with ≤50% invasive component showed MUC1+/MUC2− carcinogenetic pathway progressing to papillary/tubular adenocarcinoma, whereas a few IPNBs with ≤50% invasive progressed to mucinous carcinoma characterized by a MUC1+/MUC2+ pathway. Sasaki et al. [37] showed that the polycomb group protein enhancer of zeste homolog 2 may play a role in the regulation of MUC1 and MUC6 in IPNBs.

### 5.2. IPNB Originated from Peribiliary Glands

IPNB normally arises from the biliary epithelium in the extra- or intrahepatic large bile duct. However, recently, IPNBs that involved significantly the peribiliary glands and grossly showed cystic dilatation particularly aneurysmal or diverticular dilatation were reported [47–49], suggesting that some type of IPNB may arise from the peribiliary glands located within the wall or scattered in the surrounding connective tissue of the intrahepatic large bile ducts and extrahepatic bile ducts. These lesions are proposed to be IPNBs corresponding to pancreatic IPMN of the branch duct type [49, 50]. Sato et al. [51] showed that cystic and micropapillary changes of the epithelial cells of intrahepatic peribiliary glands, which were found in 9 (1%) of 938 autopsy livers, had abundant apical mucin and increased expression of MUC5AC, cyclin D1, and Ki-67. Since these characteristics were similar to those of pancreatic IPMN of the branch duct type, they insisted that cystic and micropapillary lesions of peribiliary glands may have neoplastic features and might represent a precursor of biliary epithelial neoplasms, including IPNB of “the branch duct type.” Cardinale et al. [52] suggested that biliary stem/progenitor cells located in the peribiliary glands might be implicated in the carcinogenesis of mucin-producing cholangiocarcinomas. However, these are still speculative.

## 6. Conclusion

Originally, IPNB was proposed as a new disease entity because of striking similarities to IPMN of the pancreas, of which the disease entity and clinicopathological features are well established. Both neoplasms share intraductal papillary growth pattern, microscopic features such as papillary proliferation with delicate fibrovascular core and 4 types of epithelial subtypes, rarely occurrence of multiple lesions, and possible progression to tubular adenocarcinoma and mucinous carcinoma. However, several important differences exist between IPNB and IPMN of the pancreas. In IPNB, pancreatobiliary type is the most common and gastric type is rare. Most cases of IPNB are IPNBs with high-grade intraepithelial neoplasia or IPNBs with an associated invasive carcinoma, and IPNBs with low- or intermediate grade intraepithelial neoplasia are infrequent. Furthermore, mucin hypersecretion is usually observed in most cases with IPMN of the pancreas, whereas only one-third of IPNB cases involve mucin hypersecretion. These differences raise a question whether all IPNBs can be included in a single disease entity. In fact, our previous study [12] revealed that IPNB without mucin hypersecretion contained heterogeneous disease groups, and the majority of IPNB without mucin hypersecretion had the characteristics close to those of nonpapillary cholangiocarcinoma. Onoe et al. [14] showed that papillary cholangiocarcinoma with >50% invasive component was clinicopathologically similar to nonpapillary cholangiocarcinoma. A lot of inconsistent data with regard to the molecular events during development and progression of IPNB mentioned above may also reflect heterogeneous disease groups in the currently defined IPNB. The concept of IPNB as a biliary counterpart of IPMN of the pancreas is attractive, but the definition of this disease entity is still somewhat confused. Further study with a large number of cases is required to elucidate the essential differences between IPNBs and BilINs.

## Figures and Tables

**Table 1:** Immunohistochemical phenotypes in intraductal papillary neoplasms of the bile duct (IPNB) and intraductal papillary mucinous neoplasms of the pancreas (IPMN) [16, 33].

Epithelial subtypes	Mucin core proteins			Cytokeratin (CK)			
	MUC1	MUC2	MUC5AC	CK20		CK7	
				IPNB	IPMN	IPNB	IPMN
Gastric	−	−	+	0 (0/5)*	0 (0/10)	100 (5/5)	80 (8/10)
Intestinal	−	+	+	75 (3/4)	71 (12/17)	50 (2/4)	82 (14/17)
Pancreatobiliary	+	−	+	22 (2/9)	0 (0/2)	78 (7/9)	100 (2/2)
Oncocytic	−~+	−~+	+	0 (0/2)	N.D.	50 (1/2)	N.D.

*% of positive cases (positive cases/total cases examined); N.D.: not determined.

**Table 2:** Molecular events in the intraductal papillary neoplasms of the bile duct lineage and the biliary intraepithelial neoplasia lineage.

Authors	Cyclin D1		p16		c-myc		*β*-catenin		SMAD4/DPC4		p53			
	IPNB	BilIN	IPNB	BilIN	IPNB	BilIN	IPNB	BilIN	IPNB	BilIN	IPNB non-inv.	IPNB inv.	BilIN non-inv.	BilIN inv.
Itatsu et al. [34]	65 (17)*	20 (45)	N.D.		54 (13)	13 (45)	22 (18)	0 (45)	N.D.		N.D.			
Nakanishi et al. [35]	53 (10)	43 (11)	N.D.		N.D.		N.D.		21 (36)	27 (49)	38 (16)	36 (10)	8 (38)	82 (11)
Schlitter et al. [36]	N.D.		24 (42)	36 (22)	N.D.		9 (45)	0 (22)	7 (45)	14 (22)	60 (52)	85 (13)	N.D.	64 (22)
Sasaki et al. [37]	N.D.		29 (34)	N.D.	N.D.		N.D.		N.D.		0 (15)	30 (19)	N.D.	
Abraham et al. [38]	N.D.		N.D.		N.D.		25 (12)	N.D.	0 (12)	N.D.	0 (12)		N.D.	

*% of cases with positive staining or mutations (total cases examined); N.D.: not determined; inv.: invasive.

**Table 3:** KRAS and GNAS mutations in the intraductal papillary neoplasms of the bile duct lineage, the biliary intraepithelial neoplasia lineage, the intraductal papillary mucinous neoplasms of the pancreas lineage, and pancreatic ductal adenocarcinoma.

Authors	KRAS mutation				GNAS mutation			
	IPNB	BilIN	IPMN	PDAC	IPNB	BilIN	IPMN	PDAC
Furukawa et al. [39]	N.D.		47 (118)*	22 (32)	N.D.		41 (118)	0 (32)
Schlitter et al. [36]	36 (45)	14 (22)	N.D.		2 (44)	0 (22)	N.D.	
Abraham et al. [38]	29 (12)	N.D.	N.D.		N.D.	N.D.	N.D.	
Matthaei et al. [40]	18 (34)	N.D.	N.D.		4 (23)	N.D.	N.D.	
Sasaki et al. [41]	46 (26)	33 (76)	N.D.		50 (30)	0 (76)	N.D.	
Tsai et al. [42]	32 (41)	N.D.	N.D.		29 (41)	N.D.	N.D.	

*% of cases with mutations (total cases examined); N.D.: not determined.
